# Barriers and Facilitators of Exercise Rehabilitation in Patients With Myocardial Infarction Based on an Updated Consolidated Framework for Implementation Research: A Systematic Review

**DOI:** 10.31083/RCM33508

**Published:** 2025-06-19

**Authors:** Ya Wang, Hualian Pei, Junjun Luo, Minfang Guan, Wenjing Sun, Hongxing Wang, Qinhong Xu

**Affiliations:** ^1^Cardiovascular Internal Medicine Ward, The Yangming Affiliated Hospital of Ningbo University, 315402 Yuyao, Zhejiang, China; ^2^Cardiovascular Internal Medicine Ward, The First Affiliated Hospital of Ningbo University, 315016 Ningbo, Zhejiang, China; ^3^Nursing Department, The First Affiliated Hospital of Ningbo University, 315016 Ningbo, Zhejiang, China; ^4^Neurosurgical Ward, The First Affiliated Hospital of Ningbo University, 315016 Ningbo, Zhejiang, China

**Keywords:** exercise rehabilitation, myocardial infarction, consolidated framework for implementation research, implementation science, systematic review

## Abstract

**Background:**

Rehabilitation through exercise is the core content of cardiac rehabilitation, which is conducive to promoting myocardial recovery and reducing mortality. However, the overall participation rate in exercise rehabilitation is low. Thus, this study aimed to comprehensively evaluate the barriers and facilitators of exercise rehabilitation for patients with myocardial infarction using the updated Consolidated Framework for Implementation Research (CFIR 2.0).

**Methods:**

Systematic research retrieval was reviewed via PubMed, Embase, Web of Science, Cochrane Library, ProQuest, and PsycINFO databases. Based on CFIR 2.0, this study used descriptive analyses to analyze the research results of each included document and identify it as a barrier or facilitator.

**Results:**

In total, 5185 studies were obtained from a preliminary search; 11 studies were ultimately included; 5 studies were quantitative. This study summarized 50 influencing factors, including 27 barriers and 23 facilitators. Most factors were related to the individual domain (64%). The remaining factors were related to the inner setting domain (20%), innovation domain (10%), implementation process domain (4%), and outer setting domain (2%).

**Conclusions:**

This study integrated the barriers and facilitators of exercise rehabilitation of patients with myocardial infarction. The study emphasizes the importance of considering the individual domain, inner setting domain, innovation domain, implementation process domain, and outer setting domain factors when implementing exercise rehabilitation. This study provides a systematic foundation for optimizing cardiac rehabilitation programs.

**The PROSPERO Registration:**

CRD42024521287, https://www.crd.york.ac.uk/PROSPERO/view/CRD42024521287.

## 1. Introduction

Myocardial infarction (MI) is an acute and critical disease of cardiovascular system caused by myocardial ischemia and hypoxia, and is associated with a high mortality [1]. With the development of interventional therapy, critical care technology and evidence-based medicine, the short-term mortality rate of patients has decreased [2]. However, the recurrence rate and long-term mortality rate of heart events are still high. Patients have health problems such as decreased activity, endurance, and excessive psychological pressure. These issues adversely affect the quality of life of patients, threatens life and health, and brings an increased economic burden to families and society.

Exercise rehabilitation, a core content of cardiac rehabilitation, is an important part of continuous care for patients with an MI [3]. Exercise rehabilitation is conducive to stabilizing, delaying or even reversing the process of atherosclerosis, promoting myocardial recovery, and reducing the mortality rate [4, 5]. Exercise rehabilitation can also help to control risk factors, improve exercise endurance and improve quality of life, which has been included as a Level I recommendation for cardiovascular disease prevention and treatment in relevant guidelines [6]. Although exercise plays an important role in the rehabilitation of patients with an MI, the overall participation rate is only 40% [7]. Research shows that in Europe, the participation rate of patients in cardiac rehabilitation is only 30%, Portugal only 8%, and in the United States it can reach 20%–30% [8]. There are many reasons for the low participation rate of cardiac rehabilitation, which may be due to the lack of rehabilitation facilities, psychological barriers, social class, and the level of education [9].

Previous studies focused on the barriers [10, 11] (anxiety, old age, diastolic dysfunction) of exercise rehabilitation, and paid less attention to the facilitators [12] (encouragement, companionship, and self-confidence). The Consolidated Framework for Implementation Research (CFIR) was first published in 2009 [13]. The primary goal of this framework is to help researchers to clarify the barriers and facilitators of the implementation process [14]. CFIR 2.0 includes five main dimensions: innovation, outer setting, inner setting, individuals, and implementation process. CFIR 2.0 is used to identify barriers and facilitators, develop implementation strategies, and evaluate the effects of implementation. However, its main orientation is still as a decisive factor framework, providing researchers with a structured method to analyze and understand various factors that affect the successful implementation of projects, policies or interventions. In order to fill the existing gaps in the implementation literature of exercise rehabilitation for patients with myocardial infarction, we systematically evaluated the barriers and facilitators of exercise rehabilitation by using the updated CFIR 2.0 [14].

## 2. Methods

### 2.1 Searches

The protocol of this systematic review has been registered in the International Prospective Register of Systematic Reviews (PROSPERO ID: CRD42024521287).

Systematic retrieval of the research on the influencing factors of exercise rehabilitation of patients with myocardial infarction was reviewed in PubMed, Embase, Web of Science, Cochrane Library, ProQuest and PsycINFO. The retrieval time is from the establishment of the database to March 2024. The retrieval method is based on the combination of subject words and free words: ① myocardial ischemia, heart infarction, heart attack*, cardiovascular stroke, acute myocardial ischemia, infarction*, myocardial, stroke*, myocardial infarct*, a non-ST-elevation myocardial infarction , ST-elevation myocardial infarction, acute coronary syndrome; ② exercise therapy, exercise rehabilitation, exercise management, remedial exercise sports, physical exertion, rehabilitation*, physical*, train*, strength*, aerobic*, exercise*, fitness, physical education; ③ barrier, facilitator, enabler, promote, drive, obstacle, encourage, hinder, discourage, workplace issues, experience, perspective, challenge.

### 2.2 Study Inclusion and Exclusion Criteria

Inclusion criteria: ① The subjects were patients with myocardial infarction over 18 years old; ② The research content was to explore the promotion, obstacles or influencing factors of exercise rehabilitation in patients with myocardial infarction; ③ The types of research are qualitative research, quantitative research and mixed research.

Exclusion criteria: ① Unable to obtain the original text; ② Repeated publication; ③ Non- English literature.

### 2.3 Study Screening and Data Extraction

After the literature was imported into endnote to remove duplicate literature, two researchers screened the literature according to the title, abstract and full text. If there was a disagreement, they discussed it with the third researcher and finally decided to include the literature. Two researchers independently extracted data, including the author, country, publication years, research design, sample size, age of subjects and data collection methods. The data extracted by the researchers are the influencing factors of the results in quantitative research and the factors mentioned in “thematic analysis” in qualitative research.

### 2.4 Study Quality Evaluation

Two researchers used the mixed methods appraisal tool (MMAT) [15], to independently evaluate the quality of the included literature. When two researchers disagreed and could not form a unified opinion after discussion, the third party’s opinion was sought and a consensus was reached after discussion with the research group.

### 2.5 Data Synthesis and Presentation

Based on the five dimensions of CFIR 2.0, this study used the descriptive analysis method to analyze the research results of each included document and identify it as either a barriers or facilitators in one of the five dimensions.

## 3. Results

### 3.1 Specification of Included Studies

**Fig. 1.**
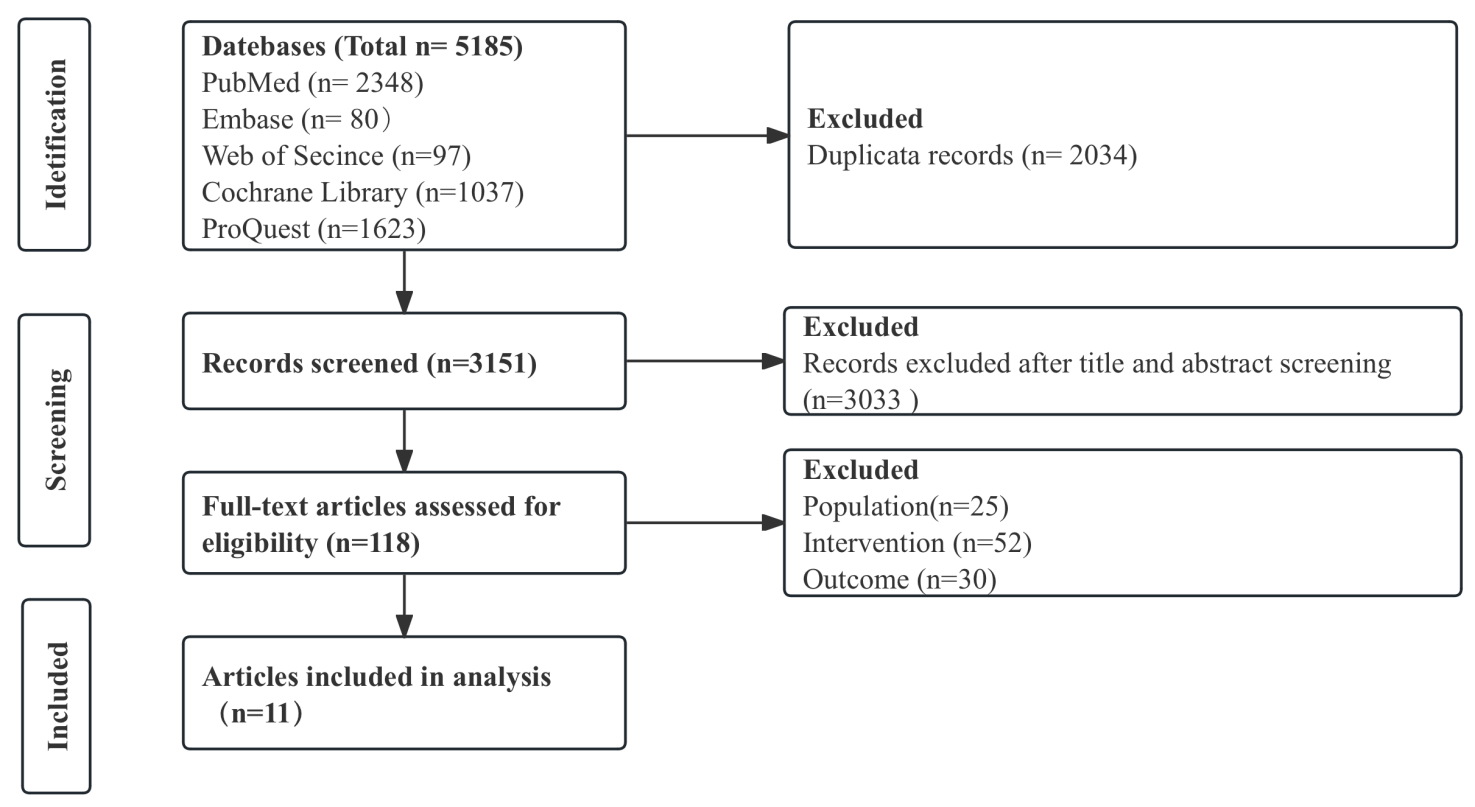
**The flow chart of the included studies**.

5185 studies were obtained from the preliminary search, and 2034 studies were excluded because of duplication. After checking the titles and abstracts, 3033 unrelated studies were excluded. 118 studies were excluded after screening the full text. 11 studies [11, 12, 16, 17, 18, 19, 20, 21, 22, 23, 24] were finally included (Fig. 1).

### 3.2 Characteristics of Included Studies

Table 1 (Ref. [11, 12, 16, 17, 18, 19, 20, 21, 22, 23, 24]) shows the characteristics of the included studies. The included studies were published in 7 countries from 1991 to 2023: Britain (n = 3), Malaysia (n = 1), China (n = 1), Jordan (n = 2), Canada (n = 2), Turkey (n = 1) and Sweden (n = 1). 6 studies [12, 16, 17, 18, 19, 20] used qualitative and semi-structured interviews, with sample sizes ranging from 8 to 21 and ages ranging from 28 to 81. 5 studies [11, 21, 22, 23, 24] were quantitative, including 4 cross-sectional studies and 1 nested case-control study. The sample size ranged from 42 to 275. Most of the participants were male.

**Table 1.** **The characteristics of the included studies**.

Author and year	Country	Aim	Study design	Participants	Sample size (n)/male (n)	Age (year)	Method of data extraction/tool
Alex Coull and Gemma Pugh, 2021 [16]	Britain	To investigate MI survivors’ attitude and appraisal towards PA and the perceived barriers, motivators and facilitators for maintaining PA long-term	Qualitative	Adults (minimum 18 years old); previous diagnosis of MI by a physician; MI occurred >5 months pre-interview; English language understood and spoken fuently; permanent address in the UK; medically ft to undertake interview.	18/13	Mean age: 60.5 years, range 37–73 years	Grounded theory methodology, semi-structured interview
Gareth Thompson *et al*., 2022 [17]	Britain	Explored the factors related to participation incardiac rehabilitation and long-term exercise from the perspectives of post-acute myocardial infarction (AMI) patients and their significant others	Qualitative	Post- AMI patients; declined or agreed to participate in a phase-III CR programme or phase-IV CR programme; sufficient English language skills to understand and participate in an interview discussion; over 18 years of age; identified significant other provides informed consent to participate in the study.	10/8	Mean age: 64 years, range 37–77 years	Semi-structured interview
Harlinna binti Abu *et al*., 2021 [12]	Malaysia	Understand how self-efficacy for physical activity is developed in a patient after MI by examining their perceptions and personal adherence to physical activity	Qualitative	Male under the age of 65; agree to participate in the study and oral consent was obtained.	8/8	Range 28–61 years	Semi-structured interview
Maria Bäck *et al*., 2020 [18]	Sweden	Explore patients’ perceptions of kinesiophobia in relation to physical activity and exercise 2 to 3 months after an acute myocardial infarction	Qualitative	A principal diagnosis of myocardial infarction; A value of ≥32 on the Tampa Scale for Kinesiophobia Heart (TSK-SV Heart).	21/13	Mean age: 64 years, range 43–81 years	Semi-structured interview
Paul M Galdas *et al*., 2012 [19]	Canada	Describe Punjabi Sikh patients’ perceived barriers to engaging in physical exercise following MI	Qualitative	19 years of age or older; diagnosed with MI in the past 12 months; able to speak Punjabi or English; self-identifying as Punjabi Sikh.	15/10	Range 48–80 years	Semi-structured interview
Sarah B Birtwistle *et al*., 2022 [20]	Britain	Explore the lived experiences of patients’ engagement with PA post-MI, together with the experiences of their family	Qualitative	≥18 years of age; an MI diagnosis within the previous month; a fluent English speaker and present in the study region for the study duration.	6/3	Mean age: 68 years, range 60–79 years	Semi-structured interview
G Godin *et al*., 1991 [21]	Canada	Understand the intention to exercise of individuals who suffer from CHD	Cross-sectional study	≤70 years; had not been hospitalized for more than 15 days (uncomplicated MI) at the time of his first myocardial infarction.	161/137	Mean age: 52.8 ± 8.1years	The item-analytic procedure suggested by Valiquette, Valois, Desharnais, and Godin (1988)
Nahla Al-Ali and Linda G Haddad, 2004 [22]	Jordan	Describes the effect of health belief model (HBM) in explaining exercise participation among Jordanian myocardial infarction patients	Cross-sectional study	Experienced first attack of MI; alert and oriented; able to ambulate.	98/57	Mean age: 50 ± 12.15 years	Health Belief Questionnaire; a self-reported questionnaire
Abedalmajeed Shajrawi *et al*., 2021 [23]	Jordan	Identify the perceived benefits and barriers to exercise and the predictors of exercise self-efficacy among patients after AMI	Cross-sectional study	Admitted to coronary care units (CCUs) with a clinically confirmed first AMI according to international criteria by European Society of Cardiology guidelines; 18 years or older and able to read, comprehend; write in Arabic; participants did not receive cardiac rehabilitation or related intervention to promote self-efficacy, health lifestyle adherence, or cardiovascular risk factor control.	254/140	Mean age: 58.5 ± 11.26 years	Exercise Self-Efficacy Questionnaire; Exercise Barriers and Benefits Scale (EBBS)
Miaomiao Du *et al*., 2023 [11]	China	Evaluate the safety of the early cardiopulmonary exercise test (CPET) and assess the predictors and clinical influence of exercise capacity measured by CPET in patients with AMI within 1 week after PCI	Nested case-control study	Age ≥18 years; first MI with definite diagnosis; successful PCI treatment, culprit vessel residual stenosis less than 20% immediately after treatment, and blood flow grade of thrombolysis in MI (TIMI) grade III after operation; completion of CPET within 1 week after PCI treatment; signed informed consent for PCI form and agreed to undergo CEPT examination and data collection.	275/253	Mean age: 58.20 ± 10.51 years	Data collection demographics, medical history, medication history, laboratory data, echocardiographic parameters, coronary angiography data and CPET parameters were collected from medical records
Hazal Yakut Ozdemir *et al*., 2023 [24]	Turkey	Explore the exercise phobia and related factors in patients with myocardial infarction	Cross-sectional study	A history of MI of between one month and one year; clinically stable health status; no change in medications over the previous three weeks; willingness to participate in the study.	42/29	Mean age: 58.38 ± 5.62 years	TSK-SV Heart, 6-minute walk test (6MWT), International Physical Activity Questionnaire- Short Form (IPAQ-SF), modified Medical Research Council (mMRC) Dyspnea Scale, Hospital Anxiety and Depression Scale (HADS), 27-item MacNew Heart Disease Health-Related Quality of Life Questionnaire

PA, physical activity; CR, cardiac rehabilitation; CHD, coronary heart disease; PCI, percutaneous coronary intervention; MI, myocardial infarction.

### 3.3 Study Quality Assessment

This study is a systematic evaluation of mixed method research. Select MMAT was used to evaluate the quality of the included study. MMAT advises against grading studies. The quality of the study included in this study varies with MMAT evaluation. Three of the six qualitative studies included in this study were limited by the lack of sufficient data to support the interpretation of the results [12] and there was inconsistency between the source, collection, analysis and interpretation of the data [12, 18, 19] (Table 2, Ref. [12, 16, 17, 18, 19, 20]). Three of the five quantitative studies included in this study were limited by whether there was complete outcome data that was not described [23] and whether confounding factors were considered in the design and analysis [22, 24] (Table 3, Ref. [11, 21, 22, 23, 24]).

**Table 2.** **Bias assessment for qualitative studies (n = 6)**.

Author and year	Are there clear research questions?	Do the collected data allow to address the research questions?	Is the qualitative approach appropriate to answer the research question?	Are the qualitative data collection methods adequate to address the research question?	Are the findings adequately derived from the data?	Is the interpretation of results sufficiently substantiated by data?	Is there coherence between qualitative data sources, collection, analysis and interpretation?
Alex Coull and Gemma Pugh, 2021 [16]	Yes	Yes	Yes	Yes	Yes	Yes	Yes
Gareth Thompson *et al*., 2022 [17]	Yes	Yes	Yes	Yes	Yes	Yes	Yes
Harlinna binti Abu *et al*., 2021 [12]	Yes	Yes	Yes	Yes	Yes	Can’t tell	Can’t tell
Maria Bäck *et al*., 2020 [18]	Yes	Yes	Yes	Yes	Yes	Yes	Can’t tell
Paul M Galdas *et al*., 2012 [19]	Yes	Yes	Yes	Yes	Yes	Yes	Can’t tell
Sarah B Birtwistle *et al*., 2022 [20]	Yes	Yes	Yes	Yes	Yes	Yes	Yes

**Table 3.** **Bias assessment for quantitative (non-randomized) studies (n = 5)**.

Author and year	Are there clear research questions?	Do the collected data allow to address the research questions?	Are the participants representative of the target population?	Are measurements appropriate regarding both the outcome and intervention (or exposure)?	Are there complete outcome data?	Are the confounders accounted for in the design and analysis?	During the study period, is the intervention administered (or exposure occurred) as intended?
G Godin *et al*., 1991 [21]	Yes	Yes	Yes	Yes	Yes	Yes	Yes
Nahla Al-Ali and Linda G Haddad, 2004 [22]	Yes	Yes	Yes	Yes	Yes	Can’t tell	Yes
Abedalmajeed Shajrawi *et al*., 2021 [23]	Yes	Yes	Yes	Yes	Can’t tell	Yes	Yes
Miaomiao Du *et al*., 2023 [11]	Yes	Yes	Yes	Yes	Yes	Yes	Yes
Hazal Yakut Ozdemir *et al*., 2023 [24]	Yes	Yes	Yes	Yes	Yes	Can’t tell	Yes

### 3.4 Barriers and Facilitators

This study summarized 50 influencing factors, including 27 barriers and 23 facilitators. Based on CFIR 2.0, it could be summarized into 5 dimensions. Most factors were related to the Individuals domain (64%). The remaining factors were related to the inner setting domain (20%), innovation domain (10%), implementation process domain (4%) and outer setting domain (2%) (Table 4, Ref. [11, 12, 16, 17, 18, 19, 20, 21, 22, 23, 24]). In this study, quantitative research focused more on socioeconomic factors, while qualitative research emphasizes psychological factors. The differences in the cultures of the included countries and health care systems also impacted rehabilitation barriers.

**Table 4.** **Barriers and facilitators of exercise rehabilitation in patients with myocardial infarction**.

Framework	Construct name		Barriers	Facilitators
Innovation domain				
	A. Innovation source		N/A	N/A
	B. Innovation evidence base		① Lack of individualized PA plan [16]	① Exercise under supervision/company [18]
			② It is difficult for patients to independently determine the safe exercise level [18, 19]	② Carry emergency medicine [18]
				③ Activities near home [18]
	C. Innovation relative advantage		N/A	N/A
	D. Innovation adaptability		N/A	N/A
	E. Innovation trialability		N/A	N/A
	F. Innovation complexity		N/A	N/A
	G. Innovation design		N/A	N/A
	H. Innovation cost		N/A	N/A
Outer setting domain				
	A. Critical incidents		Challenges related to migration [19]	N/A
	B. Local attitudes		N/A	N/A
	C. Local conditions		N/A	N/A
	D. Partnerships & connections		N/A	N/A
	E. Policies & laws		N/A	N/A
	F. Financing		N/A	N/A
	G. External pressure		N/A	N/A
		1. Societal pressure		
		2. Market pressure		
		3. Performance measurement pressure		
Inner setting domain				
	A. Structural characteristics		N/A	N/A
		1. Physical infrastructure		
		2. Information Technology infrastructure		
		3. Work infrastructure		
	B. Relational connections		N/A	N/A
	C. Communications		N/A	N/A
	D. Culture		N/A	N/A
		1. Human equality-centeredness		
		2. Recipient-centeredness		
		3. Deliverer-centeredness		
		4. Learning-centeredness		
	E. Tension for change		N/A	N/A
	F. Compatibility		N/A	N/A
	G. Relative priority		Difficulties in time management [12, 21]	N/A
	H. Incentive systems		N/A	N/A
	I. Mission alignment		N/A	Set exercise goals [16]
	J. Available resources		N/A	N/A
		1. Funding	N/A	Higher income [22]
		2. Space	Far away from forging facilities [23]	N/A
		3. Materials & equipment	① lack of exercise places [23]	N/A
			② Exercise facilities/information is inconvenient [16, 23]	
			③ Not familiar with exercise places [19]	
	K. Access to knowledge & information		N/A	① Get PA guidance from professionals [17, 22]
				② Acquire knowledge about acute myocardial infarction [12, 17]
				③ Learn to use the test scale to adjust exercise [18]
Individuals domain				
	A. High-level leaders		N/A	N/A
	B. Mid-level leaders		N/A	N/A
	C. Opinion leaders		N/A	N/A
	D. Implementation facilitators		N/A	N/A
	E. Implementation leads		N/A	N/A
	F. Implementation team members		N/A	N/A
	G. Other implementation support		① Anxiety of patients’ families about patients’ exercise [18]	① Provide social support [16, 17, 20, 23]
			② Family members of patients do not encourage exercise [20, 23]	② Exercise with peers with similar experiences [18]
	H. Innovation deliverers			
	I. Innovation recipients		① The patient is female [11]	① The patient is male [22]
			② The patient is older [11]	② Younger patients [22]
			③ Patients’ anxiety [16, 24]	③ The patient’s education level is high [22]
			④ Patients with depression [24]	④ Patients’ positive attitude [22]
			⑤ The patient has complications [11, 24]	⑤ Traumatic experience of patients with acute myocardial infarction [17]
			⑥ Symptoms of chest pain during PA and at other times [12, 16]	
			⑦ Side effects of patients’ drugs [16]	
			⑧ The side effects of patients with myocardial infarction itself [16]	
Characteristics subdomain				
	A. Need		Physical and mental adaptation difficulties [20, 21]	① Work needs [12]
				② Positive emotions brought by exercise to patients [16, 23]
	B. Capability		① Functional ability, physical activity level and HRQoL decreased [24]	① Exercise satisfies patients’ social skills [23]
			② Fatigue and weakness [19]	② Exercise improved the muscle tension and endurance of patients [23]
	C. Opportunity		N/A	N/A
	D. Motivation		① Negative emotions towards MI [16]	① Motivation to improve physical fitness immediately [16]
			② Negative emotions towards PA [16, 18, 23]	② Understand the health benefits and self-confidence of exercise after AMI [12, 17, 23]
			③ Lazy personality [12, 21]	③ Fear of recurrence of MI [12]
			④ Patients are worried that PA will cause the recurrence of MI [18]	
			⑤ Perceptual obstacle of exercise [22]	
Implementation Process domain				
	A. Teaming		N/A	N/A
	B. Assessing Needs		N/A	N/A
		1. Innovation deliverers		
		2. Innovation recipients		
	C. Assessing context		Atrocious weather [17]	Fresh air and scenery [17]
	D. Planning		N/A	N/A
	E. Tailoring strategies		N/A	N/A
	F. Engaging		N/A	N/A
		1. Innovation deliverers		
		2. Innovation recipients		
	G. Doing		N/A	N/A
	H. Reflecting & Evaluating		N/A	N/A
		1. lmplementation		
		2. Innovation		
	I. Adapting		N/A	N/A

#### 3.4.1 Dimension 1: Innovation Domain

Barriers: (1) Lack of individualized physical activity plan [16]; (2) It is difficult for patients to independently determine the safe exercise level [18, 19].

Facilitators: (1) Exercise under supervision/company [18]; (2) Carry emergency medicine [18]; (3) Activities near home [18].

#### 3.4.2 Dimension 2: Outer Setting Domain

Barriers: Challenges related to migration [19].

#### 3.4.3 Dimension 3: Inner Setting Domain

Barriers: (1) Difficulties in time management [12, 21]; (2) Distances from forging facilities [23]; (3) Lack of exercise facilities [23]; (4) Exercise facilities/information are inconvenient [23]; (5) Not familiar with exercise facilities [19].

Facilitators: (1) Set exercise goals [16]; (2) Higher income [22]; (3) Get physical activity (PA) guidance from professionals [17, 22]; (4) Acquire knowledge about acute myocardial infarction [12, 17]; (5) Learn to use the test scale to adjust exercise [18].

#### 3.4.4 Dimension 4: Individuals Domain

Barriers: (1) Anxiety of patients’ families about patients’ exercise [18]; (2) Family members of patients do not encourage exercise [20, 23]; (3) The patient is female [11]; (4) The patient is older [11]; (5) Patients’ anxiety [16, 24]; (6) Patients with depression [24]; (7) The patient has complications [11, 24]; (8) Symptoms of chest pain during PA and at other times [12, 16]; (9) Side effects of patients’ drugs [16]; (10) The side effects of patients with myocardial infarction [16]; (11) Difficulties with physical and mental adaptation [20, 21]; (12) Functional ability, physical activity level and decreased health-related quality of life (HRQoL) [24]; (13) Fatigue and weakness [19]; (14) Negative emotions towards MI [16]; (15) Negative emotions towards PA [16, 18, 23]; (16) Lazy personality [12, 21]; (17) Patients are worried that PA will result in the recurrence of MI [18]; (18) Perceptual obstacles to exercise [22].

Facilitators: (1) Provide social support [16, 17, 20, 23]; (2) Exercise with peers with similar experiences [18]; (3) The patient is male [22]; (4) Younger patients [22]; (5) The patient’s education level is high [22]; (6) Patients’ positive attitude [22]; (7) Traumatic experience of patients with acute myocardial infarction [17]; (8) Work needs [12]; (9) Positive emotions brought by exercise to patients [16, 23]; (10) Exercise satisfies patients’ social skills [23]; (11) Exercise improved the muscle tension and endurance of patients [23]; (12) Motivation to immediately improve physical fitness [16]; (13) Understand the health benefits and self-confidence of exercise after AMI [12, 17, 23]; (14) Fear of recurrence of MI [12].

#### 3.4.5 Dimension 5: Implementation Process Domain

Barriers: Atrocious weather [17].

Facilitators: Fresh air and scenery [17].

## 4. Discussion

In this systematic review, we identified 27 barriers and 23 facilitators from 11 peer-reviewed articles using CFIR 2.0. To the best of our knowledge, this systematic review is the first study to comprehensively analyze qualitative and quantitative research using CFIR 2.0, which has identified the barriers and facilitators of exercise rehabilitation in patients with a myocardial infarction.

### 4.1 Innovation Domain

Innovation: The “thing” being implemented [14]. There are three studies that mentioned how the innovation domain affected the exercise rehabilitation of patients with an MI [16, 18, 19]. It is important for patients to feel safe during exercise rehabilitation. The guidelines suggest that all patients should be provided with PA counseling in wound healing and athletic ability [25]. The determination of the exercise level is a key issue. Previous studies suggested that cardiopulmonary exercise test (CPET) can evaluate exercise intensity [25, 26]. High-intensity interval training is more effective than moderate-intensity continuous training in improving the cardiopulmonary health of patients with cardiovascular disease [27]. The basic advice is to consider moderate or moderate to high intensity areas as much as possible, and to consider different areas according to individual patient and disease characteristics.

### 4.2 Outer Setting Domain

Outer Setting: The setting in which the inner setting exists. There may be multiple outer settings and/or multiple levels within the outer setting [14]. One study reported how the outer setting domain affects the exercise rehabilitation of patients with an MI [19]. Canada has become a popular immigrant destination because of its policies and living conditions. Immigrants account for more than 20% of Canada’s total population, which is one of the countries with the highest proportion of immigrants in the world. Immigrants mainly come from Indian, China, the Philippines and other countries. For some patients, the process of immigration disrupts the original social network and limits the possibility of developing friendships, which in turn affects their chances of incorporating sports activities into their daily lives.

Medical insurance policies may also affect whether patients participate in exercise rehabilitation. Paying one’s own expenses will increase the financial burden of patients [28]. Medical insurance can cover part or all of the rehabilitation expenses and reduce the economic burden of patients. After the economic pressure is relieved, patients are more likely to stick to the rehabilitation plan. The optimization of a medical insurance policy may promote the popularization and quality improvement of rehabilitation services.

### 4.3 Inner Setting Domain

Inner setting: The setting in which the innovation is implemented. There may be multiple inner settings and/or multiple levels within the inner setting [14]. Eight studies mentioned how the inner setting domain affects the exercise rehabilitation of patients with an MI [12, 16, 17, 18, 19, 21, 22, 23]. Some patients felt “selfish” if they spend their free time on their own activity instead of their family [12]. Therefore, it is recommended that family members give more support and encouragement to patients. Exercise places/equipment/information are important for patients’ exercise. This suggests that the future community can provide more professional exercise sites and equipment to promote patients’ exercise rehabilitation. Our research results show that professional information guidance is equally important. The results of a study on patients undergoing lumbar disc surgery show that exercise in combination with information improved function [29]. Evidence emphasizes the importance of information and education in the whole health process, whether in the prevention stage, during treatment, early rehabilitation or long-term rehabilitation [30]. However, research shows that the information provided often cannot meet the needs of patients with coronary heart disease [31]. Therefore, it is particularly important to provide professional information to patients with an MI. In addition, the guidelines recommend that professionals provide consistent information [25]. Setting exercise goals and higher income may promote exercise rehabilitation of patients with an MI. It may be effective to provide help from the perspectives of economy, resources and publicity.

### 4.4 Individuals Domain

Individuals: The roles and characteristics of individuals [14]. Eleven studies mentioned how the individual domain affect the exercise rehabilitation of patients with an MI [11, 12, 16, 17, 18, 19, 20, 21, 22, 23, 24]. Our findings suggest that it is necessary to provide family and social support. Studies have shown that integrating the family into cardiac rehabilitation and social support may help facilitate PA-related interactions and promote positive engagement for patients [32, 33]. Exercise rehabilitation may create a social environment that promotes friendship, which in turn will encourage patients to exercise by enhancing fun, responsibility, and relieving their emotions by talking to their peers.

The side effects of drugs also hinder the exercise rehabilitation of patients with an MI. Statins are widely used in patients with cardiovascular diseases. These drugs may sometimes cause neuromuscular side effects. Muscle-related adverse events include spasm, myalgia, weakness, immune-mediated necrotizing myopathy, and rarely rhabdomyolysis [34]. Beta-blockers can cause myriad side effects including hypotension, dizziness, and bradycardia [35]. Antiplatelet or antithrombotic drugs can increase the risk of bleeding in patients [36]. These adverse reactions may hinder exercise rehabilitation. Clinical follow-up of patients taking these drugs by the medical staff and regular follow-up of patients may identify early side effects. An attempt should be made to better adjust drug dosages to avoid side effects.

Our results show that fear of recurrence of an MI is both a barrier and a facilitator. As a barrier, patients worry that exercise will increase the burden on the heart and lead to the recurrence of a myocardial infarction, contributing to the avoidance of rehabilitation activities. Patients may overprotect themselves, reduce necessary exercise, and delay the rehabilitation process. As a facilitator, fear of recurrence can stimulate patients to actively participate in rehabilitation, so as to reduce future health risks. Moderate worry makes patients strictly abide by the rehabilitation plan. Carrying out rehabilitation under the guidance of exercise rehabilitation professionals can reduce unnecessary worries. Through education, patients’ understanding of myocardial infarction and the rehabilitation process can be enhanced, and unknown fears can be reduced. Studies have shown that negative emotions will have a negative impact on patients with an MI and are related to poor prognosis [37, 38]. Negative emotions such as anxiety, depression and fear have greatly hindered the exercise rehabilitation of patients with an MI. Exercise-based cardiac rehabilitation can relieve anxiety and depression symptoms [39]. Appropriate psychological intervention can also reduce the negative emotions of patients with an MI [40]. Therefore, the medical staff should listen to patients’ perceptions of an MI. Psychological intervention and disease knowledge education are necessary for patients with an MI to eliminate negative psychology and promote patients’ exercise rehabilitation. Women, low education levels, and low income also hinder patients’ sports rehabilitation. The medical staff should focus on disease education for this group of patients.

### 4.5 Implementation Process Domain

Implementation process: The activities and strategies used to implement the innovation [14]. One study showed how the implementation process domain affects the exercise rehabilitation of patients with an MI [17]. In many patients, weather conditions determine the applicability of outdoor sports. If the weather is bad, this may prevent patients from going out to exercise. Medical staff or family members can encourage patients to exercise indoors.

To turn the obstacle factors in sports rehabilitation into the promotion factors, we need to adopt comprehensive strategies to help patients overcome psychological and physical obstacles and enhance their rehabilitation motivation. Psychological counseling is necessary to help patients cope with fear and anxiety. At the same time, achievable small goals should be set to enhance patients’ sense of accomplishment. Family and friends should be encouraged to participate, organize rehabilitation groups, and provide emotional support. A personalized rehabilitation plan should be formulated according to the specific needs of patients. Community and medical resources should be integrated to provide more rehabilitation support. Virtual reality technology can be used to increase the interest and interaction of rehabilitation training. Wearable devices to monitor patients’ exercise data and provide real-time feedback should be used. Through psychological support, personalized planning, social support, behavioral intervention and technical application, obstacles in sports rehabilitation can be effectively transformed into promoting factors, helping patients to better recover from their MI.

This study reviews the barriers and facilitators of exercise rehabilitation for patients with an MI based on CFIR 2.0. However, this study has several limitations. The author placed the extracted text under each CFIR 2.0 structure, based on the identified barriers and facilitators implied by the text fragments. CFIR 2.0 brings additional challenges, because researchers may encode texts in different ways. Our assessment of risk bias, and the fact that only studies that meet the standards are included, may lead to the omission of other research results. The selection of only English-language studies and the reliance on MMAT for quality assessment may introduce selection and evaluation biases. We suggest that the meta-analysis method should be used in future research to quantify the relative influence of each field factor, so as to enhance the robustness of the results. In the future, it is necessary to include more non-English studies to provide a more global perspective. Additionally, future work might incorporate a meta-analytic approach to quantify the relative impact of each domain’s factors. The CFIR 2.0 framework only provides an associative analysis and cannot determine causality. It is suggested that a prospective intervention design should be adopted in future research.

## 5. Conclusions

This study integrated the barriers and facilitators of exercise rehabilitation of patients with an MI based on CFIR 2.0. We discussed our views on these factors and possible solutions. This study emphasizes the importance of considering Individuals domain, inner setting domain, innovation domain, implementation process domain and outer setting domain factors when implementing exercise rehabilitation. These findings may provide information for future research to support the implementation of exercise rehabilitation for patients with an MI.

## Abbreviations

CFIR 2.0, the updated Consolidated Framework for Implementation Research; MMAT, the mixed methods appraisal tool; MI, myocardial infarction; PA, physical activity.

## Supplementary Material

Supplementary material associated with this article can be found, in the online version, at https://doi.org/10.31083/RCM33508.
