# Supplementary material for: Barriers and Facilitators of Exercise Rehabilitation in Patients With Myocardial Infarction Based on an Updated Consolidated Framework for Implementation Research: A Systematic Review
Source: Rev Cardiovasc Med. 2025 Jun 19;26(6):33508. doi: 10.31083/RCM33508 (PMC12230817; doi:10.31083/RCM33508)

**Pubmed 2348**

**((Myocardial Ischemia[Title/Abstract] OR heart infarction[Title/Abstract] OR heart attack*[Title/Abstract] OR cardiovascular stroke[Title/Abstract] OR Acute Myocardial Ischemia[Title/Abstract] OR infarction *[Title/Abstract] OR myocardial, stroke *[Title/Abstract] OR myocardial infarct *[Title/Abstract] OR NSTEMI[Title/Abstract] OR STEMI[Title/Abstract] OR ACS[Title/Abstract]) AND (Exercise Therapy[Title/Abstract] OR exercise rehabilitation[Title/Abstract] OR exercise management[Title/Abstract] OR remedial exercise Sports[Title/Abstract] OR Physical Exertion[Title/Abstract] OR rehabilitat*[Title/Abstract] OR physical*[Title/Abstract] OR train*[Title/Abstract] OR strength*[Title/Abstract] OR aerobic*[Title/Abstract] OR exercise*[Title/Abstract] OR fitness[Title/Abstract] OR Physical Education[Title/Abstract])) AND (barrier[Title/Abstract] OR facilitator[Title/Abstract] OR enabler[Title/Abstract] OR promote[Title/Abstract] OR drive[Title/Abstract] OR obstacle[Title/Abstract] OR encourage[Title/Abstract] OR hinder[Title/Abstract] OR discourage[Title/Abstract] OR workplace issues[Title/Abstract] OR experience[Title/Abstract] OR perspective[Title/Abstract] OR challenge[Title/Abstract])**


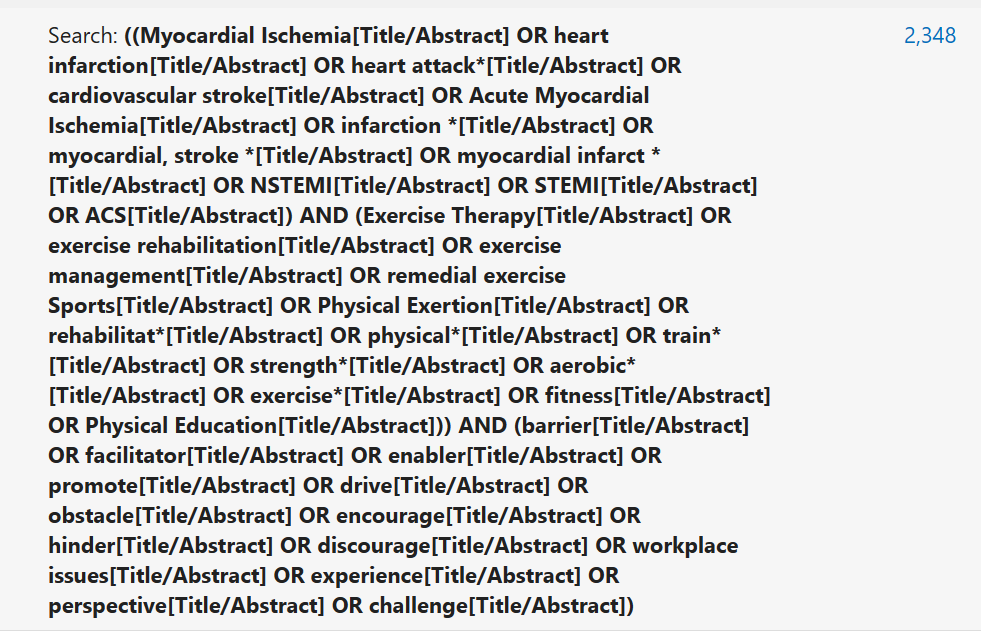


**Web of secince 97**

((KP=(Myocardial Ischemia OR heart infarction OR heart attack* OR cardiovascular stroke OR Acute Myocardial Ischemia OR infarction * OR myocardial, stroke * OR myocardial infarct * OR NSTEMI OR STEMI OR ACS)) AND KP=(Exercise Therapy OR exercise rehabilitation OR exercise management OR remedial exercise Sports OR Physical Exertion OR rehabilitat* OR physical* OR train* OR strength* OR aerobic* OR exercise* OR fitness OR Physical Education)) AND KP=(barrier OR facilitator OR enabler OR promote OR drive OR obstacle OR encourage OR hinder OR discourage OR workplace issues OR experience OR perspective OR challenge)


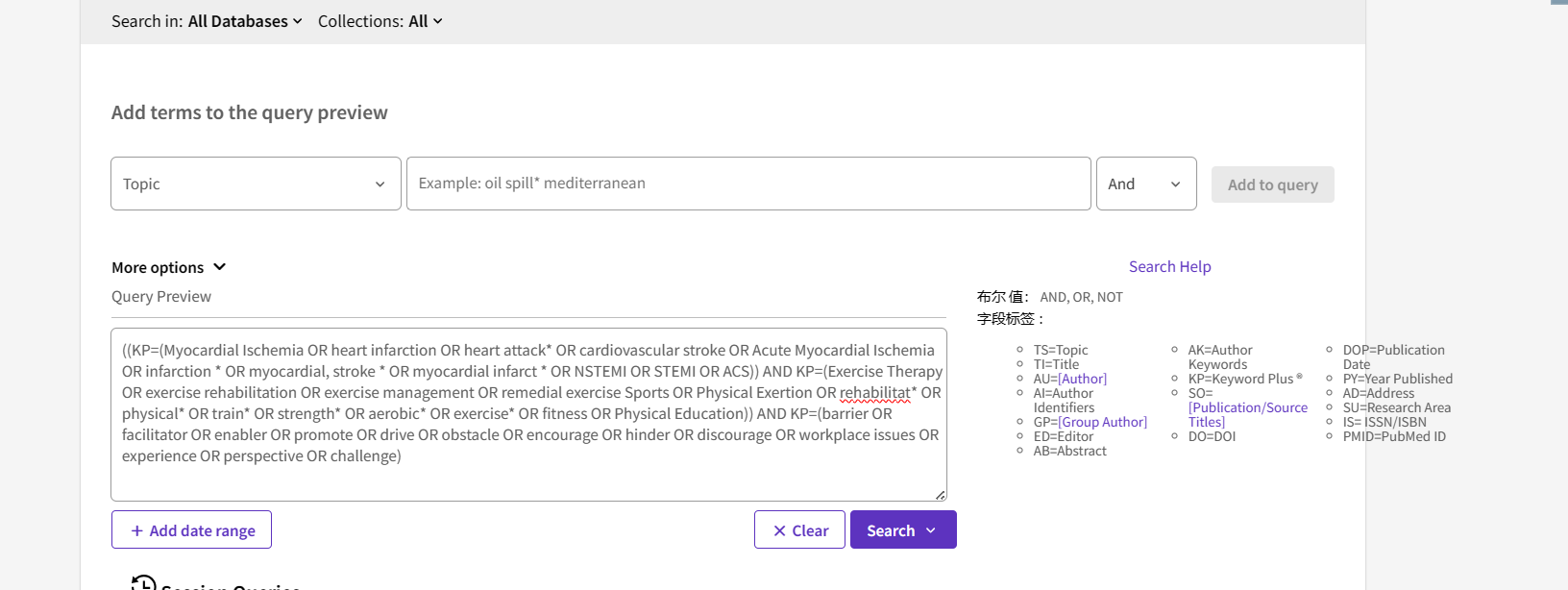


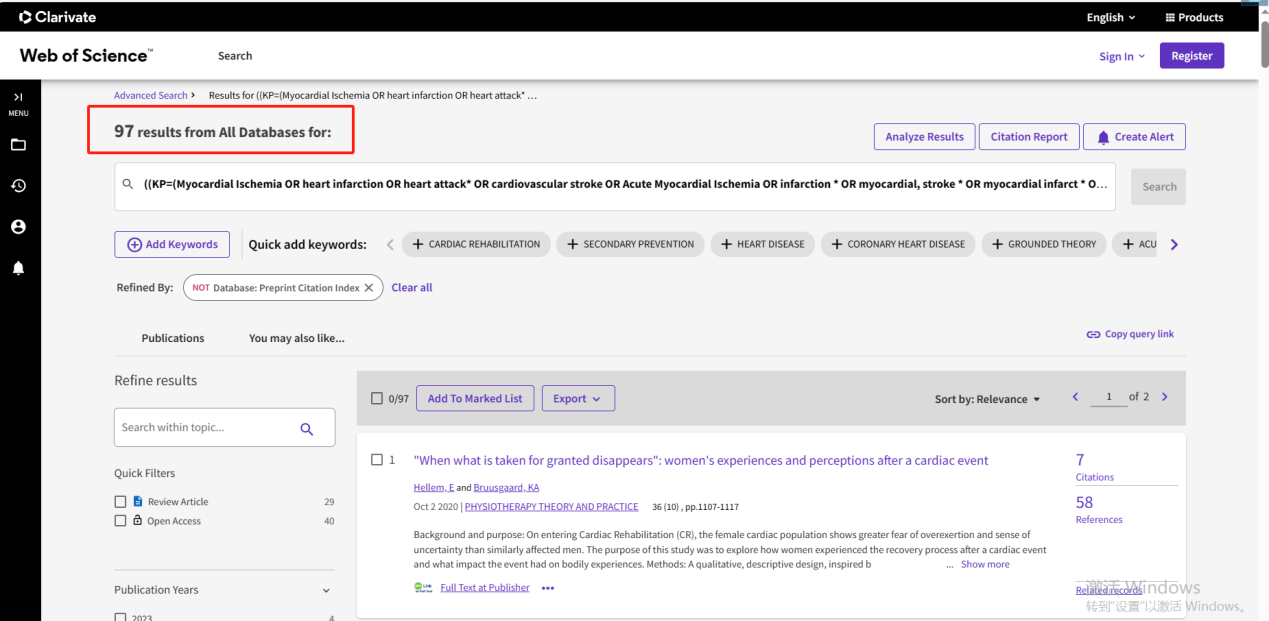


**Embase 80**

(((myocardial AND ('ischemia'/exp OR ischemia) OR 'heart'/exp OR heart) AND ('infarction'/exp OR infarction) OR 'cardiovascular'/exp OR cardiovascular) AND ('stroke'/exp OR stroke) OR acute) AND myocardial AND ('ischemia'/exp OR ischemia) AND (((((('exercise'/exp OR exercise) AND ('therapy'/exp OR therapy) OR 'exercise'/exp OR exercise) AND ('rehabilitation'/exp OR rehabilitation) OR 'exercise'/exp OR exercise) AND ('management'/exp OR management) OR remedial) AND ('exercise'/exp OR exercise) AND ('sports'/exp OR sports) OR physical) AND ('exertion'/exp OR exertion) OR 'fitness'/exp OR fitness OR physical) AND ('education'/exp OR education) AND ((barrier:ab,ti OR facilitator:ab,ti OR enabler:ab,ti OR promote:ab,ti OR drive:ab,ti OR obstacle:ab,ti OR encourage:ab,ti OR hinder:ab,ti OR discourage:ab,ti OR workplace:ab,ti) AND issues:ab,ti OR experience:ab,ti OR perspective:ab,ti OR challenge:ab,ti)


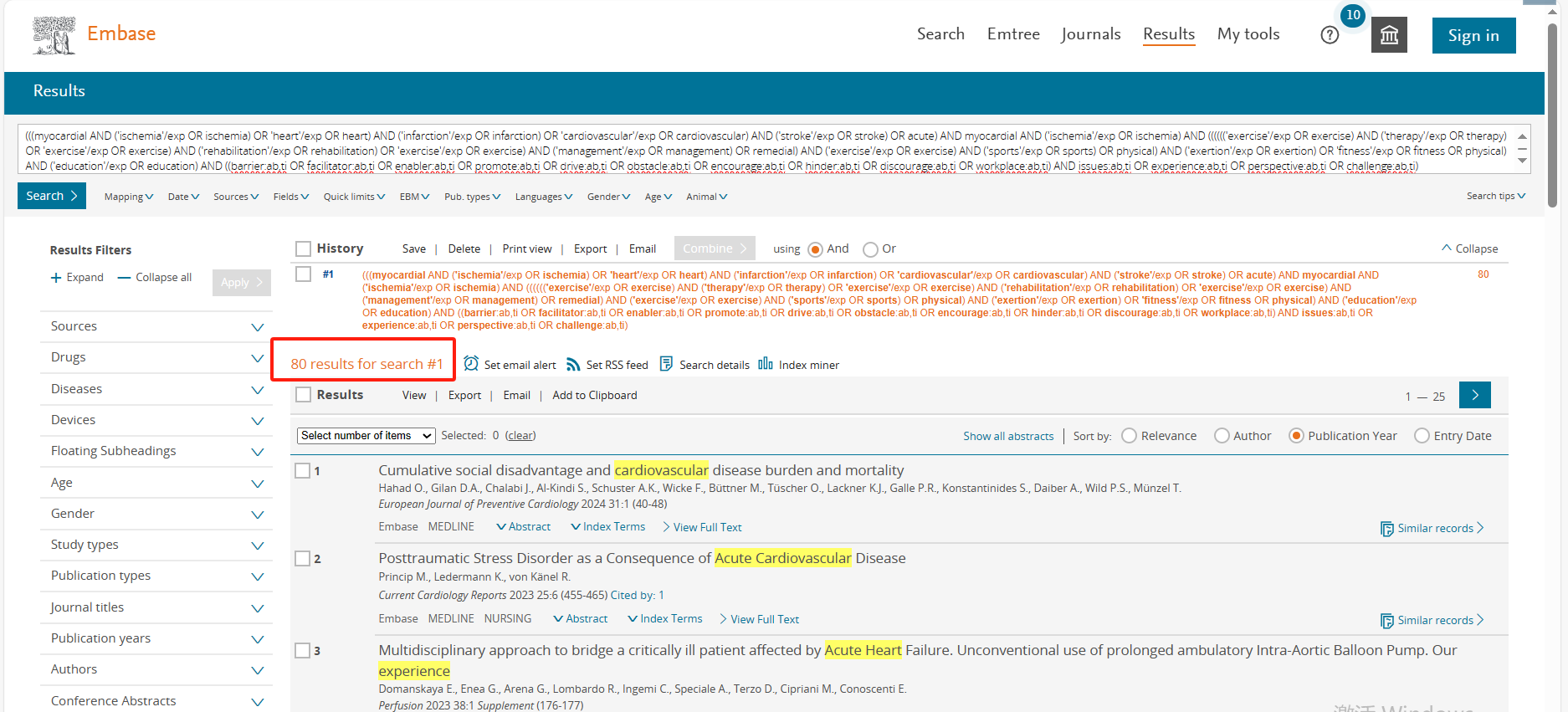


**PsycINFO 564**


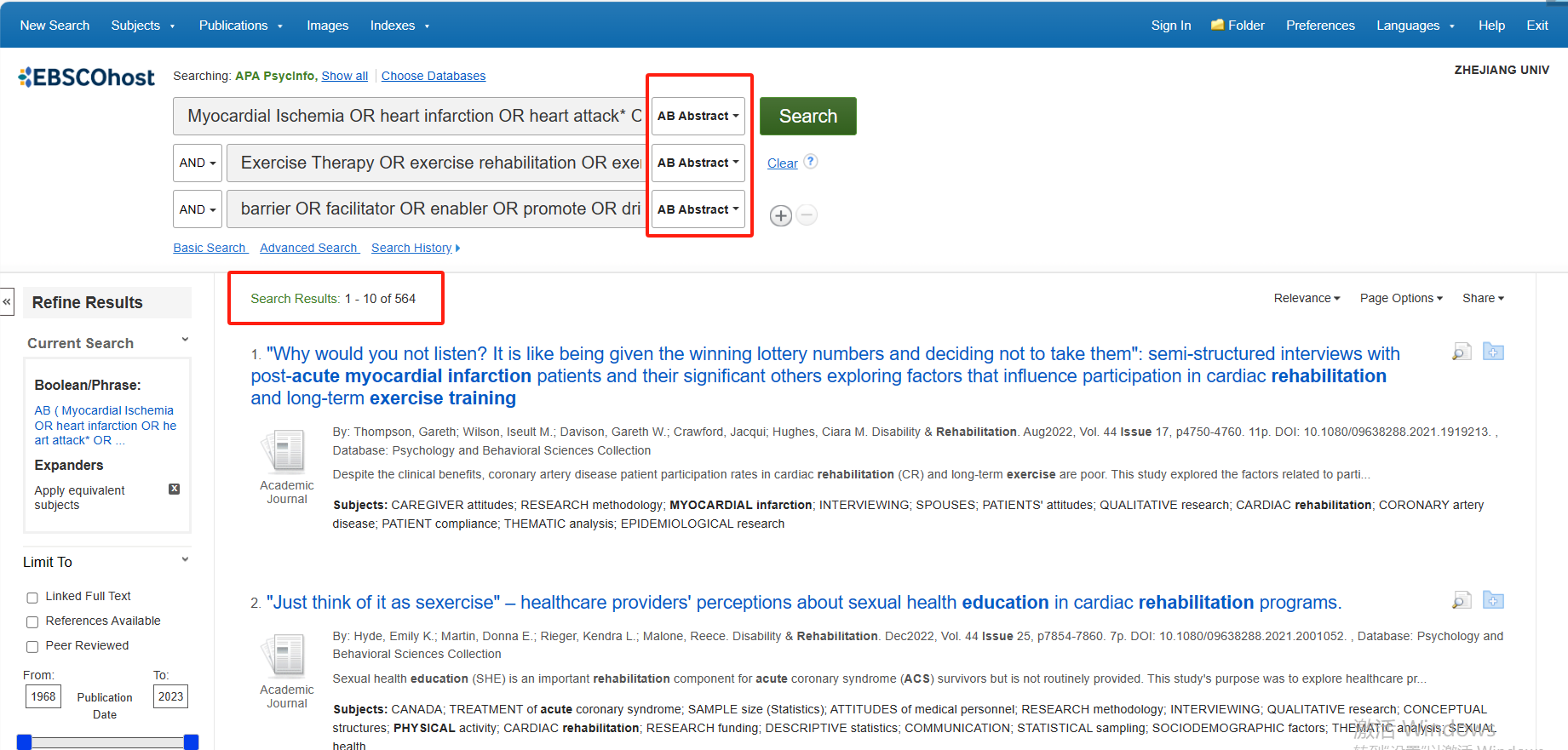


**Cochrane Library 1037**


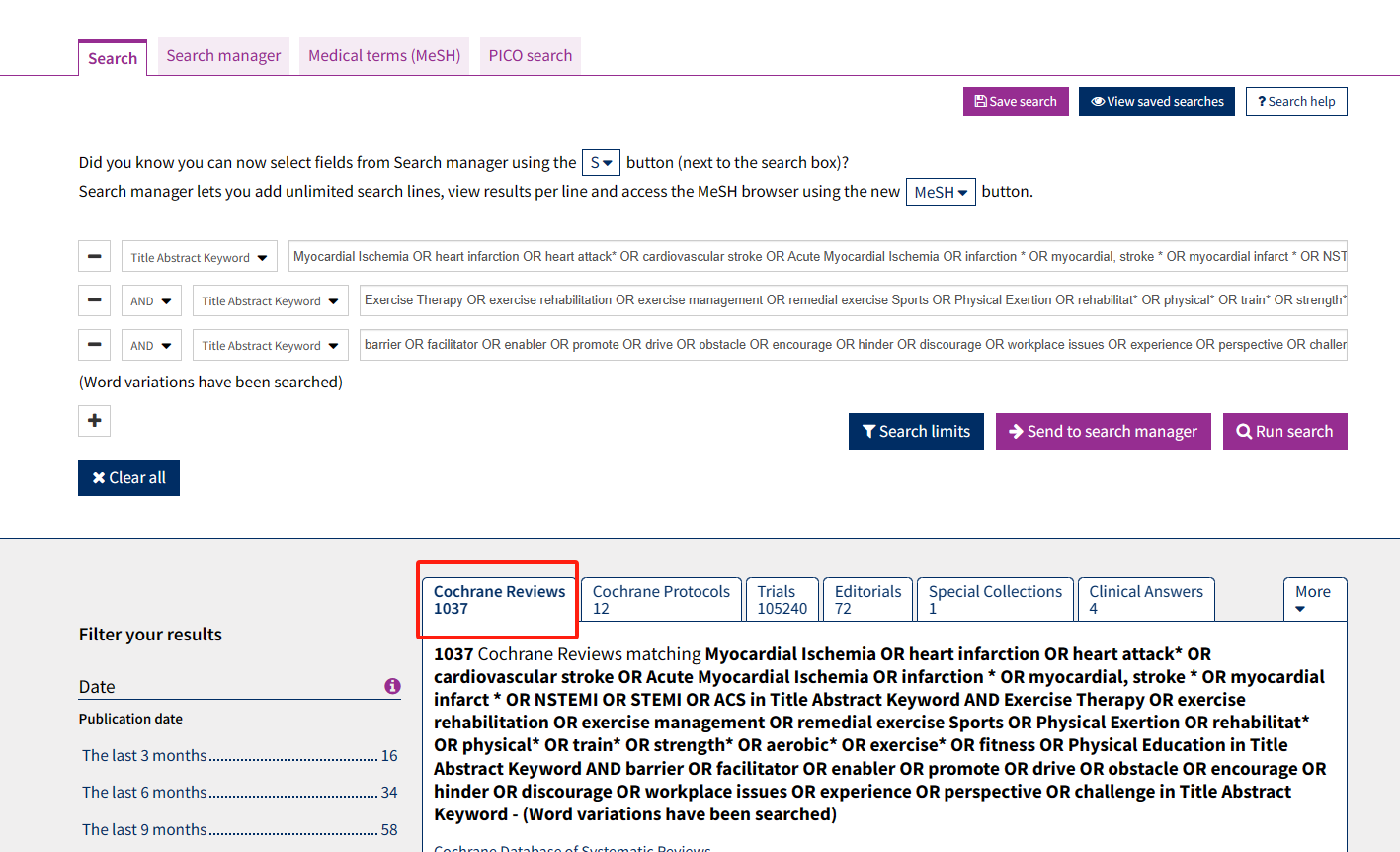


**ProQuest 1623**

summary(Myocardial Ischemia OR heart infarction OR cardiovascular stroke OR Acute Myocardial Ischemia OR NSTEMI OR STEMI OR ACS) AND summary(Exercise Therapy OR exercise rehabilitation OR exercise management OR remedial exercise Sports OR Physical Exertion OR rehabilitat* OR physical* OR train* OR strength* OR aerobic* OR exercise* OR fitness OR Physical Education) AND summary(barrier OR facilitator OR enabler OR promote OR drive OR obstacle OR encourage OR hinder OR discourage OR workplace issues OR experience OR perspective OR challenge)


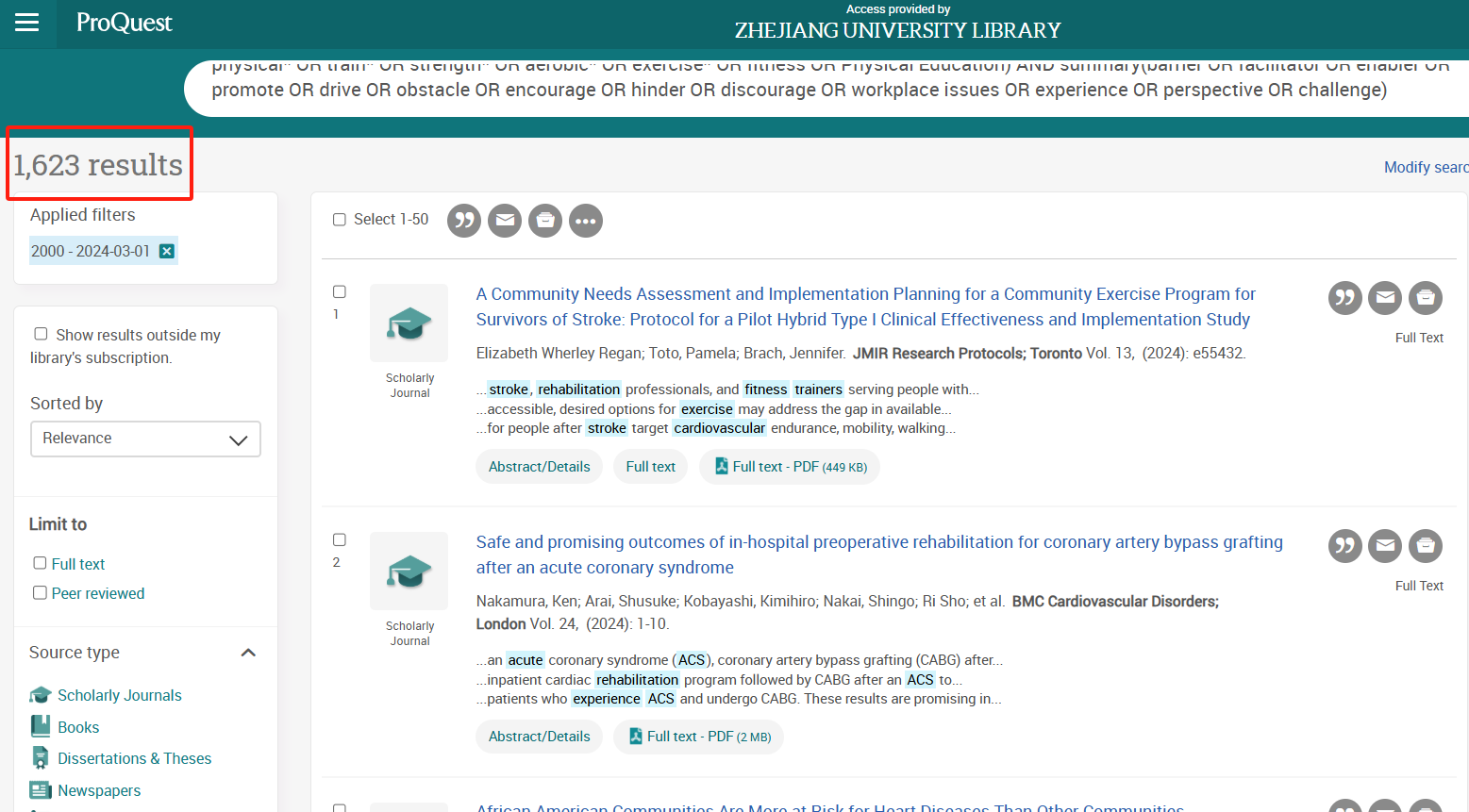

Supplement: Supplementary file 1 [file 2153-8174-26-6-33508-s1.zip › Supplementary Material S1.docx]
